# MetaIDNet: A Fast Brain–Computer Interface for Color Vision Assessment

**DOI:** 10.3390/s26196197

**Published:** 2026-09-30

**Authors:** Hadi Habibzadeh, Daphney-Stavroula Zois, James J. S. Norton

**Affiliations:** 1 The Department of Electrical and Computer Engineering, University at Albany, State University of New York, Albany, NY 12222, USA; 2 Albany Stratton VA Medical Center, Office of Research and Development, United States Department of Veterans Affairs, Albany, NY 12208, USA; 3 Albany Research Institute, Inc., Albany, NY 12208, USA; 4 Department of Psychology, Union College, Schenectady, NY 12308, USA

**Keywords:** metamer identification, Gaussian process regression, neural networks, color vision assessment, active learning

## Abstract

Our laboratory has recently demonstrated objective, quantitative, and precise brain–computer interface (BCI)-based color vision (CV) assessment. During assessment, participants look at a flashing stimulus that alternates between two light sources. Light sources that appear to be different colors elicit larger steady-state visual evoked potentials (SSVEPs); those that appear to be similar colors elicit smaller SSVEPs. Thus, the stimulus-evoked responses depend on an individual’s CV. BCI-based CV assessments evaluate CV by observing SSVEP sizes elicited over a color space; their accuracy depends on the sampling strategy and noise in the individual observations. Existing systems uniformly sample the color space and average multiple SSVEPs observed from the same location to reduce noise. Effectively, these systems collect additional observations to improve assessment accuracy, but at the expense of assessment speed. We present MetaIDNet, a closed-loop BCI that uses Gaussian process regression (GPR) and a neural network to improve the speed–accuracy tradeoff of BCI-based CV assessment. We perform offline and online experiments with ten and eight participants, respectively, to evaluate the speed and accuracy of MetaIDNet and compare it with two existing methods. Our offline experiments show that MetaIDNet improves CV assessment speed by 70% (while maintaining accuracy), and our online experiments show that MetaIDNet improves CV assessment accuracy by 40% (while maintaining the same speed). The results confirm that MetaIDNet significantly improves the speed and accuracy of BCIs for CV assessment.

## 1. Introduction

Color vision (CV) assessments are useful tools for identifying CV deficiencies, individual differences in CV, and changes in CV over time. They have traditionally been used to identify congenital and acquired CV deficiencies that affect 20% or more of the world’s population [1]. Congenital CV deficiencies can affect an individual’s ability to perform everyday tasks [2] and their career prospects [3,4,5]. Acquired CV deficiencies are potentially more common than congenital CV deficiencies and may be signs of disease or aging [6]. Beyond their use in traditional clinical applications, CV assessments also enable the study of individual differences in normal trichromatic CV, the monitoring of changes in CV over time, and changes in CV in relation to specific events [6,7].

Color-matching using an anomaloscope is the current gold-standard method for CV assessment [8]. The anomaloscope presents two light sources to the participant: a reference light source (e.g., amber light-emitting diode (LED)) and a test light source (e.g., mixture of red and green LEDs). The participant adjusts the luminances of the two light sources to make their colors match. These color matches are known as metamers—light sources with different spectral distributions that appear to be the same color. Metameric matches depend on an individual’s CV. Thus, they can be used to differentiate individuals with and without CV deficiencies, or to evaluate individual differences in normal trichromatic CV [9,10].

Despite being the gold standard, CV assessment using an anomaloscope has multiple drawbacks. The instrument is expensive, requires expertise to administer [11], and can be less effective for individuals motivated to defeat the CV test [12,13]. Additionally, it requires the participant’s active participation, reducing its usefulness for individuals with limited communication capabilities (e.g., due to cognitive impairments, motor deficits, young age, etc.) [14].

Norton et al. [15] recently proposed a brain–computer interface (BCI)-based CV assessment that does not require the participant’s active participation [15,16,17]. Their assessment evaluates changes in brain activity in response to a visual stimulus that alternates between a monochromatic (i.e., amber) reference light source and a dichromatic (i.e., mixture of red and green) test light source at a fixed rate (for the remainder of this paper, we will refer to these as the monochromatic and dichromatic light sources, respectively). If the colors of the two light sources are different, looking at the stimulus elicits a steady-state visual evoked potential (SSVEP) that can be measured using electroencephalography (EEG). The size of the SSVEP decreases the more similar in color the two light sources appear; if the colors of the two light sources are the same (i.e., they are metamers), the size of the SSVEP is minimized. Thus, analyzing the size of the elicited SSVEP can quantify how well the participant distinguishes the colors and is roughly analogous to anomaloscope-based color matching. BCI-based CV assessment has demonstrated potential for identifying people with congenital CV deficiencies [16] and may enable identification of individual differences in normal CV.

Norton et al.’s BCI-based assessment [15] evaluates CV by observing SSVEP sizes over a color space. Their strategy was to uniformly sample the color space and to average the response of multiple runs of CV assessment to reduce measurement noise. Thus, the accuracy of Norton et al.’s BCI-based assessment [15] depends on the number of unique colors of the dichromatic light source presented to each participant (i.e., sampling of the color space) and the noise in the individual SSVEP observations. Presenting more colors improves accuracy [18] but reduces the speed of BCI-based CV assessment [19,20]. Thus, for Norton et al.’s system [15], there is a tradeoff between speed and accuracy. The system can perform either fast or accurate (but not fast and accurate) CV assessment.

In this paper, we present MetaIDNet, a closed-loop BCI that improves the speed–accuracy tradeoff of BCI-based CV assessment. MetaIDNet uses a neural network to reduce the number of colors of the dichromatic light source that must be presented to each participant and Gaussian process regression (GPR) [21,22,23] to estimate the SSVEP sizes for all colors (including those that are not presented). Reducing the number of colors that must be presented to the participant increases the speed of CV assessment; estimating the SSVEP sizes with low error preserves accuracy. Our method has some analogies to the literature in adaptive psychophysics, where stimulus selection is informed by previous participant responses [24,25]. Classic and modern adaptive psychophysical methods (e.g., Quick Estimation by Sequential Testing) use closed-loop analysis to accelerate sensory threshold identification [26]; recent research in this area has even used GPR for in-home audiometry [24].

We performed offline and online experiments to evaluate the individual-level CV assessment performance (in people with normal trichromatic CV) of MetaIDNet compared with two baseline systems based on Norton et al.’s [15] work. Our offline experiments show that MetaIDNet reduces the time required for BCI-based CV assessment by 70% (while maintaining the same CV accuracy). The online experiments show that MetaIDNet can be used in real time as a closed-loop BCI to improve the accuracy of CV assessment by 40% (while maintaining the same CV assessment speed).

The rest of this paper is organized as follows: Section 2 reviews the existing methods for BCI-based CV assessment. Section 3 details the formulation of MetaIDNet and describes the neural network and GPR model. Section 4 discusses our offline and online experiments; it describes the implementation of MetaIDNet, our data collection procedures, the baseline systems, and our performance metrics. Section 5 presents the numerical results of the offline and online experiments. Section 6 discusses the results, limitations of the study, and potential directions for future work. Finally, Section 7 summarizes the study and provides concluding remarks.

## 2. Background

### 2.1. Baseline Systems

**Figure 1:**
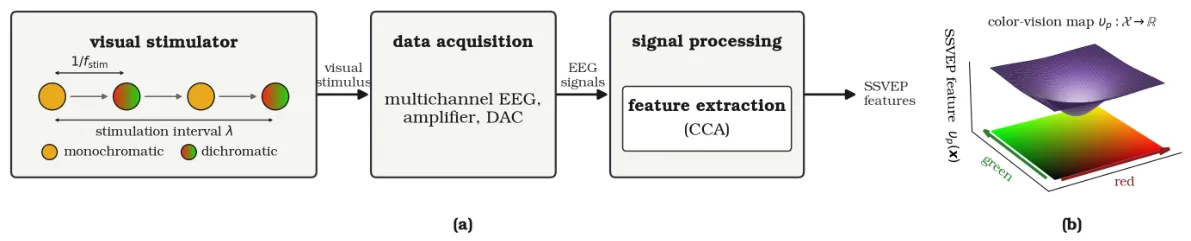
(**a**) Architecture of the BCI-based CV assessment described by Norton et al. [15]. Their system consists of three components: visual stimulation, data acquisition, and signal processing. (**b**) In this paper, we view BCI-based CV assessment as estimating the unknown function $\upsilon_{p}$ for each participant *p* that maps each color in the color space of the dichromatic light source to an SSVEP feature; $\upsilon_{p}$ characterizes the participant’s CV over the color space X.

Previous work in our laboratory (i.e., Norton et al. [15]) developed the first BCI-based CV assessment system. This system (Figure 1a) comprises three components: visual stimulation, data acquisition, and signal processing.

The visual stimulation component produces a stimulus that alternates between a monochromatic light source and a dichromatic light source at frequency $f_{\mathrm{stim}}$ for a stimulation interval of λ seconds, eliciting an SSVEP. The monochromatic light source is generated by an amber LED; the dichromatic light source is generated by two LEDs, one red and one green. During CV assessment, the luminance of the amber LED (i.e., monochromatic light source) is held constant. The luminances of the red and green LEDs, however, are adjusted independently, enabling the dichromatic light source to produce any color within a two-dimensional red/green color space, denoted by $X\subset R^{2}$.

The data acquisition component records multi-channel EEG data from the participant and performs signal amplification, digitization, and basic preprocessing (e.g., filtering and re-referencing).

The signal processing component processes the EEG data. In Norton et al.’s [15] system, the sole purpose of this component is to extract SSVEP features from EEG data. To enable later comparison to MetaIDNet, we view Norton et al.’s [15] signal processing component as containing a single module for feature extraction (Figure 1a). Norton et al. [15] used canonical correlation analysis (CCA) [27] for SSVEP feature extraction. CCA computes a linear combination of EEG channels that maximizes the correlation with a template SSVEP. In our previous work [28], we showed that replacing CCA with filter-bank canonical correlation analysis (fbCCA) [29] improves the performance of SSVEP feature extraction for BCI-based CV assessment; fbCCA creates *b* replicas of the EEG data and filters each replica independently using a bandpass filter with unique passband frequencies. Each filtered replica is processed by CCA to obtain *b* SSVEP features. The SSVEP features are then combined (e.g., using a weighted sum) to obtain an aggregate SSVEP feature.

### 2.2. Color Map Estimation

The CV of each participant *p* can be viewed as an unknown and participant-dependent function $\upsilon_{p}:X\to R$ that maps each color in the color space of the dichromatic light source to an SSVEP feature (Figure 1b). From this perspective, the goal of BCI-based CV assessment is to quickly and accurately estimate $\upsilon_{p}$. Norton et al. [15] discuss two different approaches for estimating $\upsilon_{p}$: The first approach uses exhaustive sampling of the color space of the dichromatic light source. In this case, each color in the color space is sequentially presented to the user (for λ seconds), and the size of the SSVEP elicited by each color is extracted. Exhaustive sampling leads to an accurate estimate of $\upsilon_{p}$. However, presenting every color in the color space of the dichromatic light source is time-consuming [19]. The second approach is a closed-loop BCI that uses stochastic gradient descent over the color space of the dichromatic light source to find the colors that elicit the smallest SSVEP feature, reducing the number of colors that are presented to the participant and expediting the CV assessment. This approach, however, only investigates a subset of the color space and reduces accuracy. Additionally, numerically estimating the gradients of SSVEP features at each color in the color space adds a significant sampling overhead. Norton et al. [15] collected four additional neighboring samples at each color to compute the symmetric estimate of the gradient, resulting in a 400% sampling overhead. Thus, the approaches presented by Norton et al. [15] either quickly or accurately assess CV, but not both.

## 3. MetaIDNet

**Figure 2:**
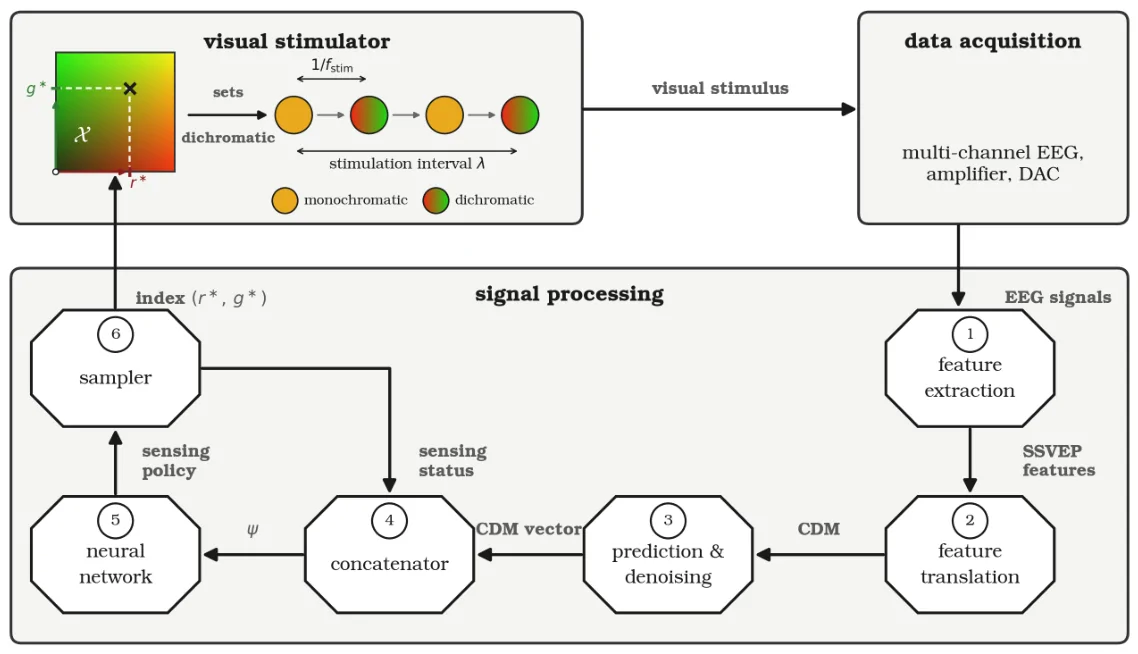
MetaIDNet is a closed-loop BCI with an enhanced signal processing component. The updated component comprises multiple modules—feature extraction, feature translation, prediction and denoising, concatenator, neural network, and sampler—designed to improve the speed–accuracy tradeoff of BCI-based CV assessment.

To improve the speed–accuracy tradeoff of BCI-based CV assessment, we introduce MetaIDNet (Figure 2). MetaIDNet is a closed-loop BCI-based CV assessment system that includes a redesigned signal processing component. The component comprises multiple modules—feature extraction, feature translation, prediction and denoising, concatenator, neural network, and sampler—designed to reduce the data required for BCI-based CV assessment. In this section, we discuss the implementation and function of each module.

### 3.1. Feature Extraction

The feature extraction module uses fbCCA [29] (Section 2) to extract *b* SSVEP features from multi-channel EEG data.

### 3.2. Feature Translation

The feature translation module, adopted from our previous work [28], converts SSVEP features $\phi \in R^{b}$ into a color-difference measurement (CDM) y∈R, where *b* is the dimensionality of the SSVEP feature space. A CDM (see Equation (1)) is obtained by performing a binary hypothesis test between the hypotheses that the colors of the two light sources are different ($H_{0}$) and the colors of the two light sources are similar ($H_{1}$), assuming that ϕ has a normal distribution under each hypothesis. Thus, a large CDM signifies that the colors of the two light sources are different, whereas a small CDM signifies that the colors of the two light sources are similar. The CDM is calculated as follows:

(1) $$\begin{array}{cc} y & =\log|\Sigma_{1}|+{\left(\phi -\mu_{1}\right)}^{T}\Sigma_{1}^{-1}\left(\phi -\mu_{1}\right)\\ & -\log|\Sigma_{0}|+{\left(\phi -\mu_{0}\right)}^{T}\Sigma_{0}^{-1}\left(\phi -\mu_{0}\right) \end{array}$$

The model parameters $\mu_{0},\mu_{1}\in R^{b}$ and $\Sigma_{0},\Sigma_{1}\in R^{b\times b}$ can be learned offline during training, as explained in Section 4.5.

### 3.3. Prediction and Denoising

The prediction and denoising module, adopted from our previous work [30], applies an interpolation filter to all observed CDMs (i.e., CDMs for colors presented to the participant) to (i) reduce noise and (ii) estimate the unobserved CDMs. In contrast to Norton et al.’s [15] exhaustive sampling strategy, the use of prediction and denoising enables MetaIDNet to observe responses for some colors and interpolate responses for others, reducing the total number of colors that have to be presented to the user during CV assessment. The interpolation filter is defined as follows:

(2) $$\begin{array}{c} \begin{array}{c} \hat{y}=Wy_{o}\\ W≜K_{o}{\left[K_{oo}+\sigma_{r}^{2}I\right]}^{-1}, \end{array} \end{array}$$

where $\hat{y}\in R^{n}$ is the estimated CDM vector corresponding to *n* unique colors in the color space of the dichromatic light source, $W\in R^{n\times n_{o}}$ is the weight matrix of the interpolation filter, and $y_{o}\in R^{n_{o}}$ is the vector of $n_{o}\leq n$ observed CDMs. $K_{o}\in R^{n\times n_{o}}$, $K_{oo}\in R^{n_{o}\times n_{o}}$, and $\sigma_{r}^{2}\in R^{+}$ are the model parameters that can be trained offline, as detailed in Section 4.5 (*I* is the identity matrix of appropriate size) [30]. In our implementation, $K_{o}$ and $K_{oo}$ are obtained by truncating $K\in R^{n\times n}$, the covariance matrix of all CDMs, and $\sigma_{r}^{2}$ is the variance of measurement noise. Thus, Equation (2) is equivalent to GPR with a constant zero mean and a covariance kernel *K*.

### 3.4. Concatenator

The *concatenator module* constructs $\psi \in R^{2n}$ as follows:

(3) $$\begin{array}{c} \begin{array}{c} \psi ≜\left[\begin{array}{c} \hat{y}\\ s \end{array}\right], \end{array} \end{array}$$

where $s≜[s_{1},s_{2},...,s_{n}]$ is a vector of *n* binary values such that $s_{i}=1$ if the *i*th color in the color space of the dichromatic light source is observed (i.e., has already been presented to the participant) and $s_{i}=0$ otherwise. The first half of ψ (i.e., $\hat{y}$) enables the neural network to use the estimated CDMs to determine the next color to present to the participant. For example, the neural network might decide to sample colors where the CDMs change abruptly, are very small, or are very large. The second half of ψ (i.e., s) enables the neural network to use the spatial distribution of previously presented colors to identify the next color to present to the participant. For example, including s in ψ enables the neural network to avoid re-sampling the same colors. Thus, ψ encapsulates the necessary information for balancing exploration and exploitation [23].

### 3.5. Neural Network

**Figure 3:**
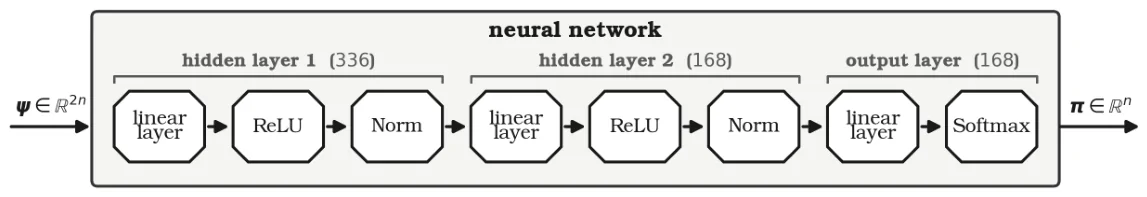
The architecture of the neural network used in the neural network module of MetaIDNet. The network is a fully connected feed-forward network with two hidden layers, each applying a linear layer followed by a ReLU activation function and batch normalization, and an output layer consisting of a linear layer followed by a Softmax function. The first hidden layer has 2n=336 units, and the second has n=168 units; the output layer has n=168 units. The network receives $\psi \in R^{2n}$ and outputs a sensing policy $\pi \in R^{n}$ that prioritizes the colors in the color space of the dichromatic light source that must be presented to each participant in real time.

The neural network module balances the tradeoff between $\hat{y}$ and s in ψ. We use a fully connected feed-forward network with two hidden layers and an output layer (Figure 3). Each hidden layer applies a linear transform followed by a rectified linear unit (ReLU) activation function and batch normalization. The output layer applies a linear transform followed by a Softmax activation function. The first hidden layer has 2n=336 units, and the second has n=168 units. The output layer has n=168 units.

The network receives ψ as the input and outputs a sensing policy $\pi =[\pi_{1},\pi_{2},\ldots ,\pi_{n}]$ such that $0\leq \pi_{i}\leq 1$ for all i∈{1,2,…,n} and $\sum_{i}\pi_{i}=1$. If $\pi_{i}>\pi_{j}$, the network *predicts* that the constructed CDM vector after sampling the *i*th color in the color space of the dichromatic light source is closer to the ground truth than the constructed CDM vector after sampling the *j*th color.

### 3.6. Sampler

The sampler module determines the next color $x_{\mathrm{next}}$ in the color space of the dichromatic light source to sample, such that

(4) $$\begin{array}{c} \begin{array}{c} x_{\mathrm{next}}=X_{i^{*}}\\ i^{*}=\arg\max_{1⩽i⩽n}\left[\pi_{i}\right], \end{array} \end{array}$$

where $X\in R^{n\times 2}$ is the matrix of all colors in the color space of the dichromatic light source, and $X_{i^{*}}$ is the $i^{*}$th element in X. After selecting $x_{\mathrm{next}}$, the sampler module sets the corresponding element in s to 1.

### 3.7. MetaIDNet

Algorithm 1 shows the application of MetaIDNet as a closed-loop BCI for CV assessment. MetaIDNet always starts by sampling the first location in the color space X (i.e., where the dichromatic light source is switched off) to obtain a baseline for CDMs (this baseline is not necessary for the operation of the algorithm but may be useful for interpreting the results). MetaIDNet keeps acquiring additional samples until a stopping criterion is met (e.g., until a certain number of samples are collected or updating the estimated CDM vector results in small changes). Acquiring additional samples improves the accuracy but reduces the speed of the BCI.
**Algorithm 1** MetaIDNet**Input:** the color space X of the dichromatic light source, sensing budget λ, inter-stimulus interval $\lambda_{\mathrm{ISI}}$, model parameters for (1) and (2)**Output:** Estimated CDM vector $\hat{y}$  1:$\hat{y}=0$  2:$x_{\mathrm{next}}=X_{0}$ (i.e., start with the first color in X)  3:s=[1,0,0,...,0]  4:**while** the stopping condition is not met **do**  5:   Turn on the stimulator for λ seconds with the color of the dichromatic light source set to $x_{\mathrm{next}}$. Collect EEG signals and extract SSVEP features.  6:   y= use (1) to extract the CDM  7:   $\hat{y}=$ update the CDM vector using (2)  8:   $\psi =\mathrm{concatenate}\;\hat{y}$ and s  9:   π= input ψ to the network to get the sensing policy10:   i= the index of the largest element in π11:   $s_{i}=1$12:   $x_{\mathrm{next}}=X_{i}$13:   Turn off the stimulus for $\lambda_{\mathrm{ISI}}$ seconds14:**end while**15:return $\hat{y}$

## 4. Materials and Methods

### 4.1. Participants

Fourteen healthy individuals (S1–S14, 18+ years old) who self-reported no neurological disorders or CV deficiencies participated in our studies. Ten individuals (S1–S10, four females) participated in our offline experiments, and eight individuals (S1, S6, S7, S9, S11–S14, two females) participated in our online experiments. All experiments were approved by the Albany Stratton VA Medical Center (SVAMC) Institutional Review Board (IRB).

### 4.2. Experiments

#### 4.2.1. Offline Experiments

Our offline experiments consisted of five runs of data collection per participant (seven and three runs of data were collected for S3 and S7, respectively). During each run, participants completed a sequence of trials that sampled 168 unique colors in the color space of the dichromatic light source (i.e., n=168). Each trial consisted of six seconds of stimulation (i.e., $\lambda_{\mathrm{offline}}=6$ s) followed by a 0.5 s inter-stimulus interval (ISI) (i.e., $\lambda_{\mathrm{ISI}}=0.5$ s). Both light sources were turned off during the ISI. The color space of the dichromatic light source X was defined as follows:

(5) $$\begin{array}{c} X≜r\times g,\\ r≜\{0,24,48,\ldots ,312\}\;\mathrm{ADU},\\ g≜\{0,12,24,\ldots ,132\}\;\mathrm{ADU}, \end{array}$$

where × is the Cartesian product and ADU is the analog-to-digital converter unit of the LED drivers that specifies the luminance of each LED.

#### 4.2.2. Online Experiments

The online experiments evaluated the closed-loop performance of MetaIDNet and consisted of twenty runs of data collection. Each run consisted of 98 trials (i.e., $n_{o}=98$; ∼5 min). Informal analyses in our lab have found that most participants can maintain their attention to the stimulus for five minutes without requiring a break. Each trial presented one unique color (in X) to the participant and lasted for three seconds—2.5 s of stimulation (i.e., $\lambda_{\mathrm{online}}=2.5$ s), followed by a 0.5 s ISI (i.e., $\lambda_{\mathrm{ISI}}=0.5$ s). The colors and their presentation order were determined by MetaIDNet in real time and varied from run to run and participant to participant.

### 4.3. Visual Stimulation

We elicited SSVEPs using the same visual stimulator as Norton et al. (detailed in [15]). The stimulator consisted of multiple LEDs, a projection system (i.e., lens and diffusion film with a 6 cm in diameter viewing port), and 3D-printed housing. The participant was asked to observe the stimulus by looking at the viewing port. The luminance of the monochromatic light sources was held constant at 600 ADU, whereas the luminances of the two LEDs used to generate the dichromatic light source varied from trial to trial. The visual stimulator was programmed to alternate between the two light sources every 50 ms (i.e., $f_{\mathrm{stim}}=10$ Hz).

### 4.4. Data Acquisition

We used a g.USB EEG amplifier (g.tec medical engineering GmbH, Schiedlberg, Austria) to collect 16 channels of EEG data from electrodes at locations PO1, O1, Oz, O2, PO8, P5, PO3, POz, PO4, P6, P3, P1, Pz, P2, P4, and CPz, per the 10–10 international system [31], at a sampling rate of 256 samples per second. A reference electrode was placed at location Cz, and a ground electrode was placed on the left earlobe (some runs of data collection in our offline experiments used the left mastoid for the ground electrode). A 12th-order IIR bandpass filter with zero phase shift was used to filter data between 5 Hz and 55 Hz.

### 4.5. MetaIDNet Implementation

#### 4.5.1. Training and Cross-Validation

Data collected during the offline experiments was used to train MetaIDNet’s signal processing component. To minimize overfitting, all of our evaluations of MetaIDNet—for both the offline and online experiments—used leave-one-out cross validation. For participants who did not complete the offline experiments, we used the signal processing component trained on data from participants S02–S10 (i.e., the model was trained excluding data from participant S01).

#### 4.5.2. Feature Extraction Module

We used fbCCA to extract SSVEP features [29] (see Section 2) from the EEG data. Our implementation of fbCCA included eight bandpass filters (i.e., b=8), where the passband frequencies of the *i*th filter were i×5 and 55 Hz, i=1,2,…,8. For example, the passband frequencies for the second filter were 10 Hz and 55 Hz. We used four harmonics to construct the template SSVEP [27].

#### 4.5.3. Feature Translation Module

We extracted *b* SSVEP features for 9.5 Hz ($\phi_{9.5}$), 10 Hz ($\phi_{10}$), and 10.5 Hz ($\phi_{10.5}$) for every trial in the training dataset. Extracted features were natural log-transformed to better satisfy the multivariate normality assumption (for validation of this assumption, see our prior work [28] and Appendix C). We estimated $\mu_{0}$ and $\Sigma_{0}$ using the sample mean and sample covariance matrix of log-transformed $\phi_{10}$. We estimated $\mu_{1}$ using the sample mean of $\log(0.5\times (\phi_{9.5}+\phi_{10.5})\;/\;1000)$ (corresponding to using small non-zero SSVEP features for $H_{1}$) and estimated $\Sigma_{1}$ using the sample covariance matrix of log-transformed $0.5\times (\phi_{9.5}+\phi_{10.5})$. Finally, we set all non-diagonal elements in $\Sigma_{0}$ to zero.

#### 4.5.4. Prediction and Denoising Module

We augmented the training data in two ways to train the prediction and denoising module: First, we used a one-second sliding window with 0.75 s overlap to compute 20 CDM vectors for each run of data collection. Second, we replaced each CDM vector with n=168 replicas, where the *i*th replica was constructed by circularly shifting the CDM vector by *i* values. Data augmentation ensured that all colors in the color space of the dichromatic light source were equally likely to match the color of the monochromatic light source (as perceived by the participant) and eliminated prior assumptions about the participant’s CV. For each participant *p* in our training dataset, we computed the sample covariance matrix of CDM vectors to obtain $K_{p}$ and estimated *K* by averaging all $K_{p}$s. The model parameter $K_{o}$ in (2) can be estimated by dropping the columns of *K* that correspond to unobserved colors. Similarly, the model parameter $K_{oo}$ in (2) can be estimated by dropping the rows and columns of *K* that correspond to unobserved colors. Finally, we used the largest element in *K* as our estimate of noise variance $\sigma_{r}^{2}$ in (2).

#### 4.5.5. Concatenator Module

The concatenator module does not require training; it concatenates the output of the prediction and denoising module with the sensing status vector s to construct ψ.

#### 4.5.6. Neural Network Module

We created a dataset D with more than two million data points for training and validation of the neural network module. Each data sample consisted of a pair of ψ and its corresponding sensing policy π, i.e.,

(6) $$\begin{array}{c} \left\{\psi ,\pi \right\}=\left\{\left[\begin{array}{c} \hat{y}\\ s \end{array}\right],\pi \right\}. \end{array}$$

Each sensing status s was constructed by randomly selecting *p* from the set {0.1,0.2,…,0.9} and drawing a random binary sequence with length *n* such that p(s=0)=p∀s∈s. Next, we selected a random CDM vector y (for a random run of a random participant) and discarded any y∈y for which the corresponding s∈s was zero to obtain the observed CDM vector $y_{o}$. Finally, we passed the observed CDM vector to the denoising and prediction module to compute the estimated CDM vector $\hat{y}$.

Each $\pi_{j}\in \pi$ for j=1,…,n was computed as follows: If the *j*th color in the color space of the dichromatic light source was observed (i.e., $s_{j}=1$), we set $\pi_{j}$ to an arbitrary negative number smaller than −1, discouraging the neural network from recommending an already-presented color. Otherwise, if the *j*th color in the color space of the dichromatic light source was not observed (i.e, $s_{j}=0$), we extended the observed CDM vector to $y_{o}\cup \left\{y_{j}\right\}$ and passed it to the denoising and prediction module to obtain ${\hat{y}}_{j}^{\mathrm{extended}}$. Next, we computed $d_{j}≜{∥{\hat{y}}_{j}^{\mathrm{extended}}-y^{*}∥}_{2}^{2}$, where ${∥\cdot ∥}_{2}^{2}$ was the square of the $\ell_{2}$-norm and $y^{*}$ was the ground-truth CDM vector (i.e., the average CDM vectors of all runs for the selected participant). If $d_{j}<d_{k}$ for some *j* and *k*, then the *j*th color in the color space of the dichromatic light source had to be sampled before the *k*th color because it led to a smaller reconstruction error. Thus, for all *j*s for which $s_{j}=0$, we can set $\pi_{j}$ based on $d_{j}$. We normalized all $d_{j}$s for such *j*s to take values in [0,1] and set $\pi_{j}=-d_{j}$. Finally, after computing $\pi_{j}$s for all *j*s, we passed the constructed π to a Softmax function.

We used sliding windows and circular shifts to augment our training dataset, as explained in Section 4.5.4. Additionally, we further augmented our data by combining CDM vectors from different runs of different participants to create combined CDM vectors. These data augmentation techniques reduced the chance of overfitting the neural network [32,33] (see Appendix E).

**Figure A1:**
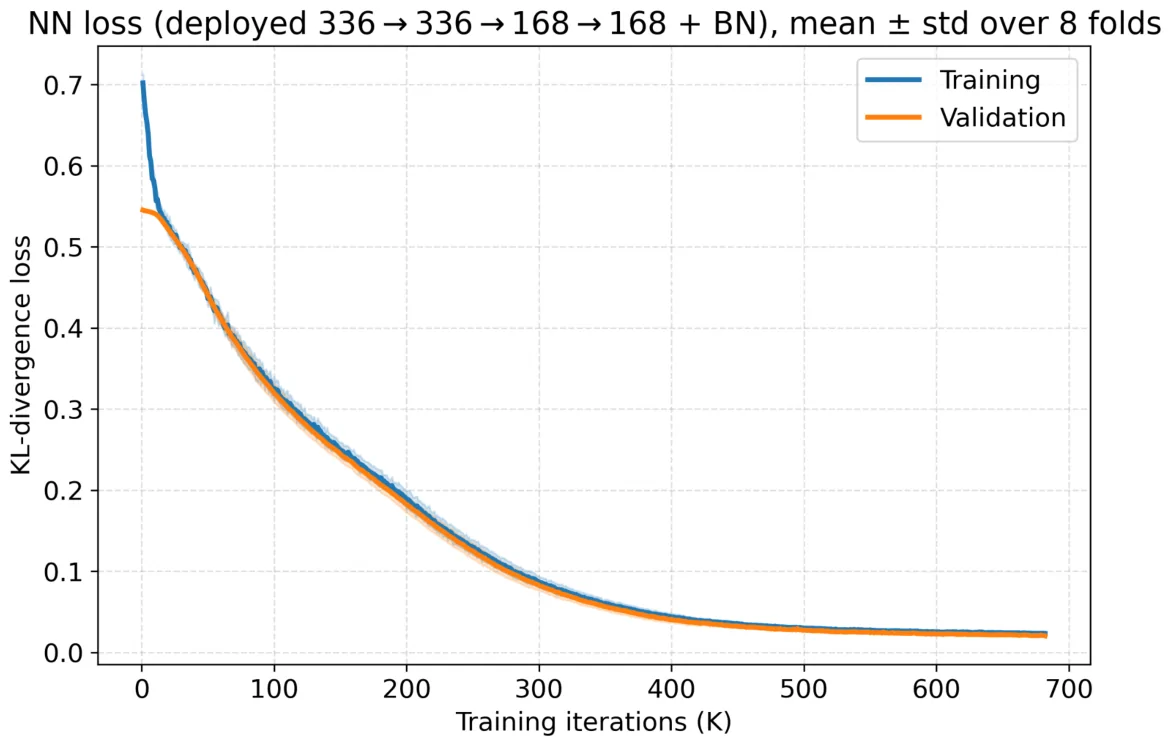
Kullback–Leibler divergence (KL) loss as a function of training iteration for the training and validation sets of the NN. The network was trained with stochastic gradient descent (SGD) (learning rate 0.1, momentum 0.9) minimizing a KL loss (batchmean, log-target, i.e., nn.KLDivLoss (reduction = “batchmean’’, log_target = True)), with validation loss logged every 1000 iterations (mini-batch size of 500). Note that the loss values logged during the original training run were not retained; the reported curves were regenerated by retraining the same network architecture with the identical training procedure and data, so they reflect the model’s training behavior rather than the exact original run. Both losses decrease smoothly, and the validation loss converges without rising, indicating stable training and no overfitting. The validation loss tracks slightly below the training loss because the training loss is averaged over each logging window (a running mean over a model that is still updating), while the validation loss is evaluated at the window’s end with batch normalization in inference mode.

Dataset D was used to train the fully connected feed-forward network (Figure 3) with two hidden layers (336 and 168 units, each with ReLU and batch normalization) and a 168-unit output layer followed by a Softmax function. The network was trained by minimizing the KL divergence loss between its output and the sensing policies in D (see Figure A1).

#### 4.5.7. Sampler Module

The sampler module selects the color with the largest corresponding π∈π to be the next color presented to the participant; it does not require training.

### 4.6. Baseline System Implementations

We implemented Norton et al.’s BCI-based CV measurement system [15] (shown in Figure 1) as our first baseline system. Additionally, we implemented an fbCCA-based system that is similar to Norton et al.’s [15] but uses fbCCA for SSVEP feature extraction; fbCCA is a common feature extraction method and is often used as a baseline in many SSVEP-based BCI publications.

### 4.7. Ground Truth and Performance Metric

The ground-truth SSVEP measurements $y_{i,j}^{*}$ (CDMs for MetaIDNet and features for the baseline systems) are the average, over all runs of participant *j*, of the measurements obtained by system *i* by exhaustively sampling every color at the full stimulation interval. This definition applies identically to all three systems (Norton et al. [15], fbCCA-based, and MetaIDNet). For MetaIDNet specifically, the ground truth is built from the CDM, i.e., the output of the feature translation module (Equation (1)), and its estimate ${\hat{y}}_{i,j}$ is the subsampled and interpolated reconstruction; MetaIDNet’s subsampling, denoising, and interpolation contribute only to the estimate under evaluation and play no role in forming the ground truth. After computing the ground truth, we define the sum of squared errors (SSE) of system *i* and participant *j* as

(7) $$\begin{array}{c} {\mathrm{SSE}}_{i,j}={∥y_{i,j}^{*}-{\hat{y}}_{ij}∥}_{2}^{2}, \end{array}$$

where $y_{i,j}^{*}$ and ${\hat{y}}_{ij}$ are the ground-truth and estimated SSVEP measurements, respectively, for system *i* and participant *j*. Finally, to enable comparison of the systems, the SSEs were z-score-standardized.

Similarly, the ground-truth SSVEP measurements (CDM vectors for MetaIDNet and SSVEP features for the baseline systems) for the online experiments were the average SSVEP measurements for all online runs of each participant. No exhaustive-sampling runs were collected online. Thus, this reference is necessarily estimated from the same online assessments. In Appendix G, we verify that our conclusions are unchanged when the online ground truths are instead evaluated against the independent offline exhaustive ground truth for the participants who attended both experiments. During the online experiments, MetaIDNet presented 98 unique (but variable from run to run and participant to participant) colors in the color space of the dichromatic light source and interpolated the CDMs for the remaining 70 colors. The CDM vectors for MetaIDNet were computed online to evaluate MetaIDNet’s performance as a real-time and closed-loop BCI for CV assessment. The baseline systems, however, are open-loop systems. Thus, the SSVEP features (the baseline systems do not use CDM vectors) for the baseline systems were computed by offline processing of the EEG data collected during our online experiments. The baseline systems did not perform any interpolation. Thus, we merged data from multiple runs of data collection for each participant to complete the final SSVEP features for the baseline systems. In computing the SSE, we discarded any portion of the SSVEP measurements that was not presented in any of the twenty runs. For all of our participants, we discarded fewer than ten samples (out of 168). Finally, to keep the assessment duration at five minutes, we used the first 1.25 s of EEG data in each trial for computing the SSVEP features for the baseline systems.

## 5. Results

### 5.1. Offline Experiments

**Figure 4:**
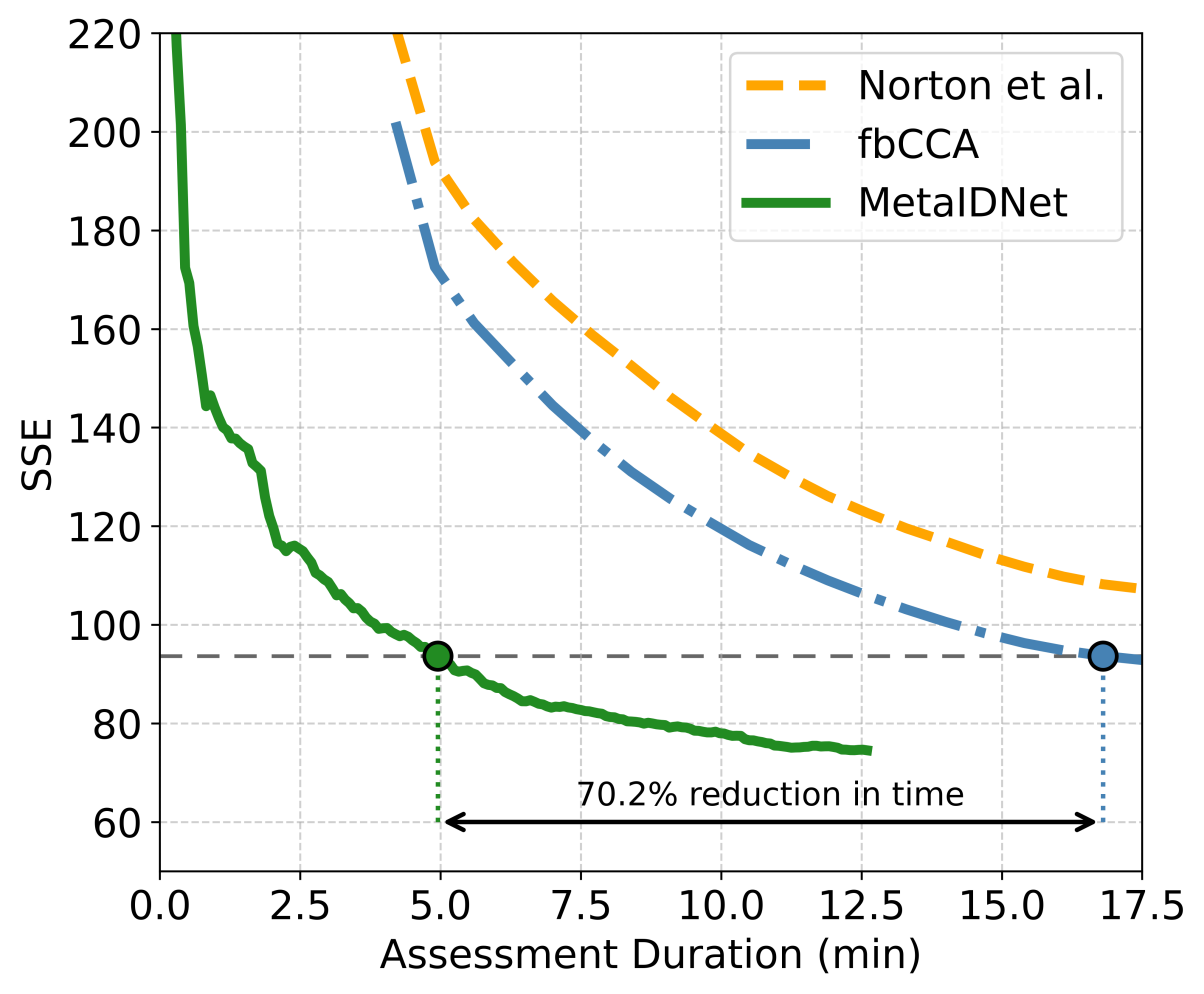
Results of the offline experiments. SSE, averaged across participants, is plotted as a function of assessment duration for MetaIDNet and the two baseline systems (i.e., Norton et al. [15] and fbCCA-based). The dashed lines illustrate that MetaIDNet only requires 5 min to achieve the same SSE (i.e., assessment accuracy) that the fbCCA-based system achieves in 16.8 min (a 70.2% reduction in time).

**Figure A5:**
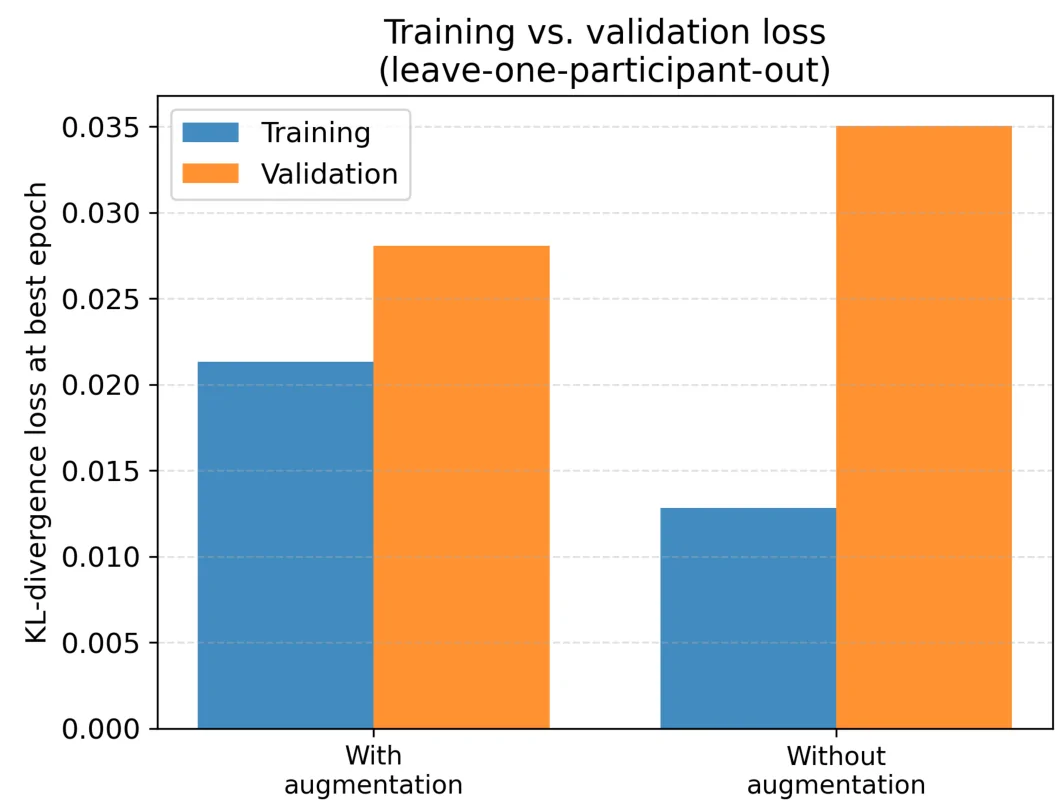
Effect of data augmentation on generalization of the NN using data from the offline experiments. Using data augmentation reduces the validation loss.

**Figure A6:**
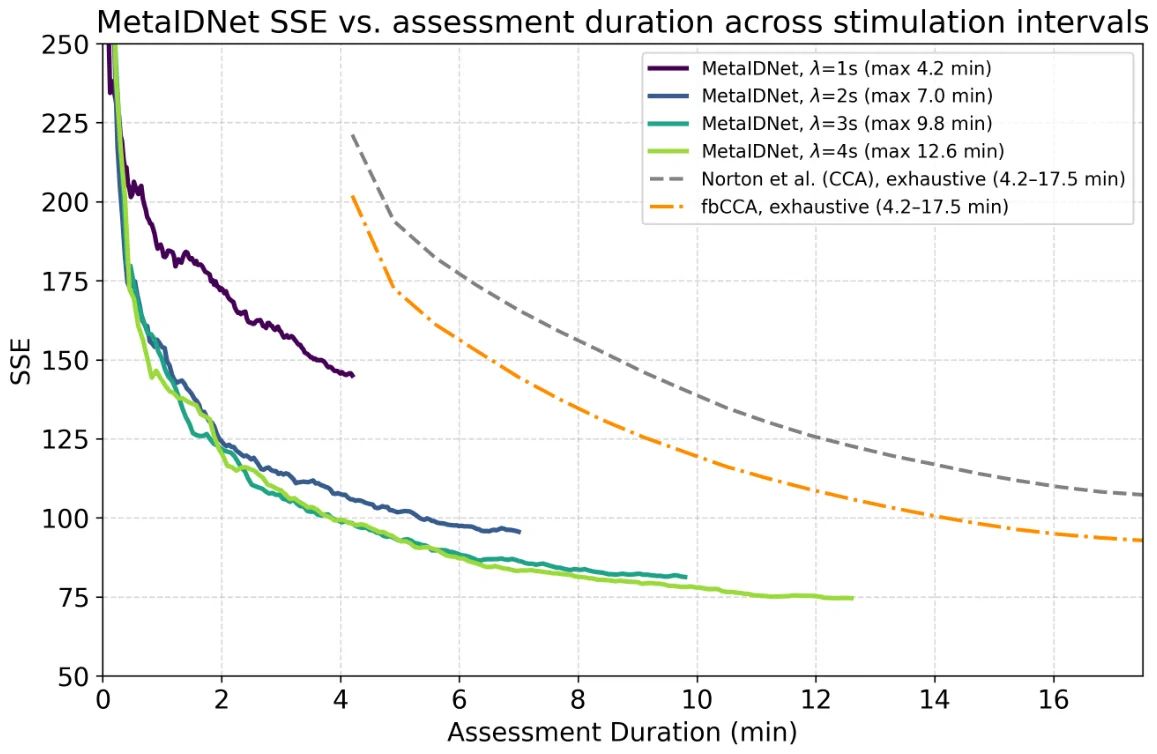
MetaIDNet’s SSE-vs-assessment-duration curves for λ = 1, 2, 3, and 4 s. Because each color is presented for λ + $\lambda_{\mathrm{ISI}}$ seconds, the stimulation interval sets both the per-color cost and the maximum achievable assessment length; for the full 168-color grid, these correspond to total durations of 4.2, 7.0, 9.8, and 12.6 min, respectively (reported in the figure legend).

Figure 4 compares the SSE of MetaIDNet and the baseline systems (following z-score standardization) as a function of assessment duration. The assessment duration of the baseline systems depends on the stimulation interval λ and ISI $\lambda_{\mathrm{ISI}}$; it is computed as $168\times (\lambda +\lambda_{\mathrm{ISI}})$, where λ∈{1,1.25,…,5.75} s and $\lambda_{\mathrm{ISI}}=0.5$ s. Thus, the assessment duration for the baseline systems varied from 4.2 min (see Table 1) to 17.5 min (see Table 2). The assessment duration of MetaIDNet is defined as $n_{o}\times \left(\lambda +\lambda_{\mathrm{ISI}}\right)$, where $n_{o}$ is the number of observed colors in the color space of the dichromatic light source. To reduce clutter, Figure 4 only shows MetaIDNet’s performance for λ=4 s (for additional values of λ, see Figure A6; for a component-level analysis (i.e., ablation test) of MetaIDNet, see Appendix D; for the generalization gap with and without data augmentation, see Figure A5). The shortest assessment duration for MetaIDNet was 4.5 s (when only one color in the color space of the dichromatic light source was observed, i.e., $n_{o}=1$). The longest assessment duration for MetaIDNet was 12.6 min (when all colors in the color space were observed, i.e., $n_{o}=168$).

The SSE of MetaIDNet was lower than that of either of the baseline systems for the shortest and longest assessment durations where direct comparisons were possible. For the shortest comparable assessment duration (4.2 min), MetaIDNet’s SSE of 98 (λ=4 and $n_{o}=56$) was lower than that of either of the baseline systems (Norton et al.’s [15] SSE = 221, t(9) = 10.53, p<0.001; fbCCA-based SSE = 201, t(9) = 10.14, p<0.001; paired *t*-tests). For the longest comparable assessment duration (12.6 min), MetaIDNet’s SSE of 74 (λ=4 and $n_{o}=168$) was, again, lower than that of both baseline systems (Norton et al.’s [15] SSE = 122, t(9) = 4.01, p=0.003; fbCCA-based SSE = 122, t(9) = 3.75, p=0.005; paired *t*-tests). Figure 4 shows that, on average, MetaIDNet required an assessment duration of 5.0 min (equivalent to observing 66 colors) to achieve the same SSE as the fbCCA-based system with an assessment duration of 16.8 min—a 70.2% reduction in the duration of BCI-based CV assessment.

### 5.2. Online Experiments

Table 3 presents the SSE for each system (following z-score standardization) and participant in our online experiments (the assessment duration was five minutes for all of the online experiments [Section 4.2]). Across participants, the mean SSE of MetaIDNet was 77, lower than that of either of the baseline systems (Norton et al.’s [15] SSE = 165, t(7) = 9.76, p<0.001; fbCCA-based SSE = 129, t(7) = 6.49, p<0.001; paired *t*-tests).

## 6. Discussion

MetaIDNet is a new closed-loop BCI that uses a neural network and GPR to improve the speed–accuracy tradeoff of BCI-based CV assessment. Compared to the fbCCA-based baseline system, MetaIDNet improves CV assessment speed by 70% (while maintaining accuracy; offline experiments) and CV assessment accuracy by 40% (while maintaining the same speed; online experiments). In this section, we describe the advantages of MetaIDNet, discuss the limitations of the present study, and make recommendations for future research on BCI-based CV assessment.

The speed of BCI-based CV assessment depends on the number of colors of the dichromatic light source presented to the participant and the stimulation interval λ. SSVEPs emerge about 120 ms after the onset of the stimulus, setting a lower bound $\lambda_{\min}$ on the stimulation interval [34] (in practice, even the heavily trained state-of-the-art algorithms for SSVEP detection require at least 0.5 s of stimulation [35]). MetaIDNet, however, only presents $n_{o}\leq n$ colors to the participant, reducing the effective lower bound on each color in the color space to $\left(n_{o}\;/\;n\right)\times \lambda_{\min}$ seconds. MetaIDNet also reduces the total ISI time. ISIs provide a momentary break from visual stimulation for the participant. However, they do not provide any additional information about the participant’s CV. MetaIDNet reduces the number of colors presented to the participant, decreasing the total ISI time by $\left(n-n_{o}\right)\times \lambda_{\mathrm{ISI}}$ seconds. Lastly, while computationally complex, MetaIDNet does not add additional computational overhead to the experiments (see Appendix C). Reducing the number of colors presented to the participant becomes more important in future BCIs for CV assessment that may include higher-dimensional color spaces (e.g., by adding a blue LED for diagnosing tritanopia), as the number of colors increases exponentially with the dimensionality of the color space. Thus, even the lower bound on the duration of CV assessment for the existing methods might become too long for practical application of future systems. MetaIDNet’s approach to subsampling the color space without reducing the accuracy might be an effective method to keep future BCIs for CV assessment fast enough for practical applications.

MetaIDNet’s performance can be further improved in three ways: First, MetaIDNet can be modified to use dynamic stimulation intervals (i.e., different stimulation intervals for different colors in the color space) instead of (or in addition to) subsampling the color space. The neural network can also be modified to predict the stimulation interval for each color. For example, the first colors presented to the participant and the colors that are expected to have small CDMs (and, hence, a small signal-to-noise ratio) can use longer stimulation intervals. Second, we used three data augmentation techniques to reduce the chance of overfitting the model (Section 4.5). However, our data augmentation method creates many CDM vectors that are unrealistic. Collecting more data and training MetaIDNet with more realistic CDM vectors could further improve its performance. Third, we trained the GPR model and the neural network separately and as independent modules. Collecting more data would enable us to train these two modules simultaneously or combine them into a single network, which may further improve the performance of the signal processing component.

Some of the design and analysis choices that we made in evaluating MetaIDNet and the two baseline systems could have an impact on the results. The ground truth used to evaluate accuracy is an estimate of each participant’s response function rather than an externally validated reference. Offline, it is obtained from exhaustive sampling at a full stimulation interval and is independent of MetaIDNet’s subsampling and reconstruction. Online, however, no exhaustive-sampling runs were collected, so the reference was estimated from the same online assessments used for evaluation. We recomputed the online sum of squared errors against the independent offline exhaustive reference for the participants who completed both experiments. While the difference in SSE between fbCCA and MetaIDNet was reduced from 58 to 28 (based on data from four participants), MetaIDNet remained the lowest-error method in every case (Appendix G). For each system, $y^{*}$ is the average of the system’s SSVEP measurements exhaustively sampled over the color space (CDMs for MetaIDNet and features for the baselines). Averaging SSVEP measurements over runs reduces noise. Thus, our accuracy metric (defined in Equation (7)) quantifies how well a system constructs its own mean response from limited data, not how closely that response matches the participant’s true CV. Because all participants had normal CV, this ground truth also characterizes normal CV. Relating MetaIDNet’s reconstructed response maps to clinically validated references in color-vision-deficient populations is an important direction for future work (discussed below). In addition, our comparisons of the three systems for the online fixed assessment speed used two different SSVEP window sizes (2.5 s for MetaIDNet and 1.25 s for the baseline models). The final result of a 40% increase in accuracy for a fixed assessment time may have been affected by this design choice.

The experiments described in the present paper have limitations. There is minimal reporting and no analysis of differences in BCI-based CV responses across age, sex, and other demographics. There are known differences in CV due to sex and aging. Assessment of differences in BCI-based CV assessment for these groups is an excellent direction for future work. Our categorization of participants as having normal CV relied on self-reporting. While most participants were aware of whether they had a congenital CV deficiency, confirmation using a standard behavioral test (e.g., the Ishihara plate test) would have improved our experiments. There were some differences in the location of the ground electrode in some of the offline data. The electrode was occasionally placed on the left mastoid instead of the left earlobe. This difference may have had an effect on the results. We expect these differences to be small given the following: (1) the purpose of the ground electrode is to reduce common-mode voltage to prevent amplifier saturation (theoretically, the ground does not contribute to EEG measurements, i.e., [electrode − GND] − [reference − GND] = electrode − REF]); (2) the small distance between the electrode locations; (3) the large distance between the left earlobe/mastoid and the occipital region where SSVEPs are most prevalent; and (4) any differences should affect MetaIDNet and the baseline systems equally. However, the differences and limited reporting of the differences are a limitation of the present paper.

Finally, in future work, we recommend the validation of MetaIDNet in multiple CV assessment applications. Recently collected data from our laboratory has demonstrated BCI-based CV assessment for the identification of red–green CV deficiencies [16]. For people with these types of CV deficiencies, MetaIDNet could be used to speed up the assessment or to help differentiate people with mild versus more severe deficiencies. In addition, the present results suggest that MetaIDNet can reconstruct the SSVEP elicited across the color space for people without CV deficiencies. Experiments could be devised to analyze relationships between these response maps and the results of standard CV assessments (e.g., anomaloscope match point). These are just two examples of many exciting potential applications for this technology.

## 7. Conclusions

BCI-based CV assessments are precise and easy to use; they only require a participant to look at a visual stimulus that alternates between a monochromatic light source (with a fixed color) and a dichromatic light source (with varying colors). Looking at the stimulus elicits a large SSVEP if the colors of the light sources are different and a small SSVEP if the colors of the light sources are similar. The speed and accuracy of BCI-based CV assessment depends on the number of colors of the dichromatic light source presented to the participant. Presenting more colors improves accuracy but reduces the speed of assessment. In this paper, we presented MetaIDNet—a closed-loop BCI that uses a neural network and GPR model to improve this speed–accuracy tradeoff. Offline experiments with ten participants showed that MetaIDNet improves CV assessment speed by 70% while maintaining a similar accuracy, and online experiments with eight participants showed that MetaIDNet improves accuracy by 40% while maintaining the same speed. In summary, MetaIDNet enables fast and accurate BCI-based CV assessment.

## Figures and Tables

**Table 1:** Offline experiments—SSE for MetaIDNet and the baseline systems for a 4.2 min CV assessment.

Participant	Norton et al.	fbCCA-Based	MetaIDNet
S01	213	207	146
S02	259	268	170
S03	190	175	84
S04	246	192	61
S05	155	129	56
S06	196	188	87
S07	202	162	90
S08	238	203	91
S09	271	280	116
S10	241	216	81
Mean ± SD	221 ± 34 *	202 ± 43 *	98 ± 34

Per-participant SSE for the offline experiments (4.2 min assessment time). The bottom row of the table reports the mean and standard deviation across the ten participants. MetaIDNet’s SSE is significantly lower, as denoted by *, than that of both baseline systems (Norton et al., t(9) = 10.53, p<0.001; fbCCA-based, t(9) = 10.14, p<0.001; paired *t*-tests). Note: Five runs of data were collected per participant. However, participants S3 and S7 completed seven and three runs, respectively.

**Table 2:** Offline experiments—SSE for MetaIDNet and the baseline systems for a 12.6 min CV assessment.

Participant	Norton et al.	fbCCA-Based	MetaIDNet
S01	123	146	118
S02	197	174	105
S03	63	70	74
S04	116	100	55
S05	61	48	41
S06	79	61	60
S07	121	86	60
S08	128	97	74
S09	182	154	85
S10	158	124	73
Mean ± SD	123 ± 44 *	106 ± 40 *	75 ± 22

Per-participant SSE for the offline experiments (12.6 min assessment time). The bottom row of the table reports the mean and standard deviation across the ten participants. MetaIDNet’s SSE is significantly lower, as denoted by *, than that of both baseline systems (Norton et al., t(9) = 4.01, p=0.003; fbCCA-based, t(9) = 3.75, p=0.005; paired *t*-tests). Note: Five runs of data were collected per participant. However, participants S3 and S7 completed seven and three runs, respectively.

**Table 3:** Online experiments—SSE for MetaIDNet and the baseline systems.

Participant	Norton et al.	fbCCA-Based	MetaIDNet
S01	149	134	75
S06	126	106	66
S07	207	179	135
S09	217	184	87
S11	161	79	58
S12	128	106	61
S13	207	154	92
S14	124	88	46
Mean ± SD	165 ± 37 *	129 ± 38 *	77 ± 26

Per-participant SSE for the online experiments. The bottom row of the table reports the mean and standard deviation across the eight participants. MetaIDNet’s SSE is significantly lower, as denoted by *, than that of both baseline systems (Norton et al., t(7) = 9.76, p<0.001; fbCCA-based, t(7) = 6.49, p<0.001; paired *t*-tests).

**Figure A2:**
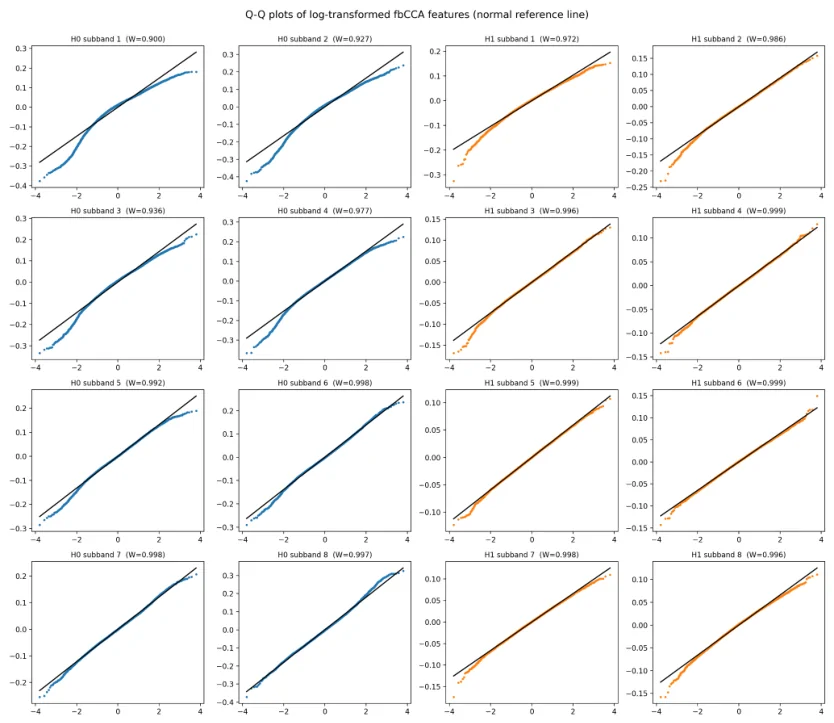
Normal Q-Q Plots comparing the log-transformed fbCCA features for each of the eight subbands under $H_{0}$ (blue; left two columns) and $H_{1}$ (orange; right two columns). The black lines show the normal reference lines and the titles show the Shapiro-Wilk statistic *W* (W=1 for exactly normal data).

**Figure A3:**
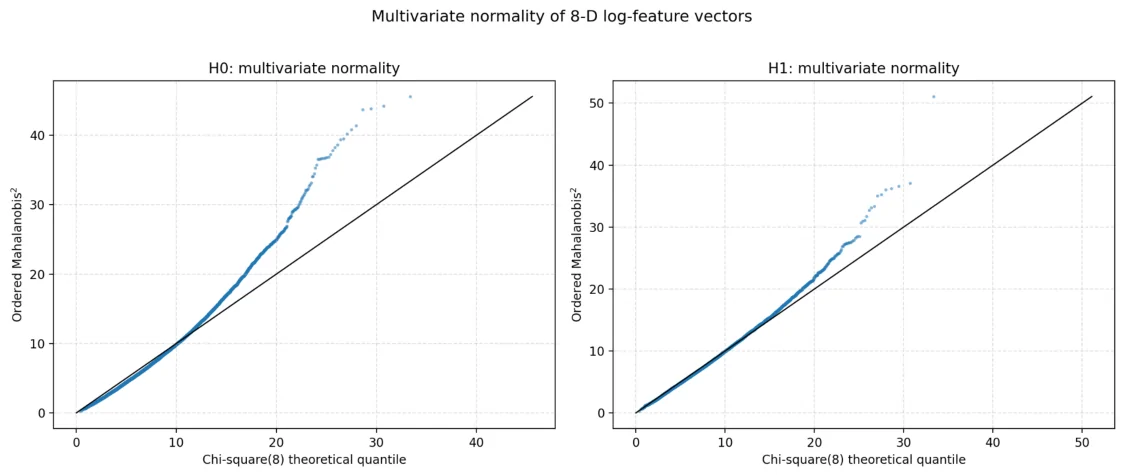
Multivariate normality of the 8-dimensional log feature vectors (if ϕ is multivariate normal, its squared Mahalanobis distance $d^{2}=\phi^{⊤}\Sigma^{-1}\phi$ follows $\chi_{8}^{2}$ quantiles). The distributions under each hypothesis follow the reference closely but with heavier tails than normal.

**Figure A4:**
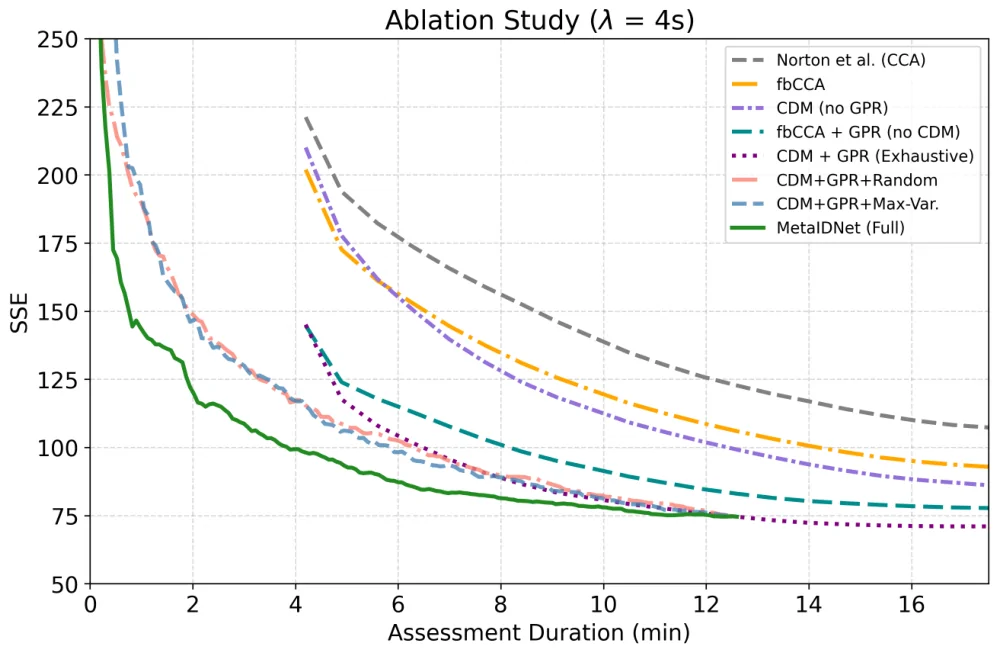
Module-level ablation study to isolate the contribution of each component to MetaIDNet’s overall performance.

## Data Availability

Data necessary for replicating this work will be made available at https://github.com/bci-assessment-lab, accessed on 18 August 2026.

## Appendix A. Neural Network Validation Performance

Data Partitioning—We used leave-one-out cross-validation to train the neural network (NN). Each held-out participant’s data was the test set, entirely excluded from the training dataset D. Within D, we split by data-collection run (samples from a participant’s last run were held out for validation), giving a 67.6%/32.4% train/validation split.

## Appendix B. Computational Complexity

GPR is a computationally expensive operation. However, at the scale of our experiment, even in the worst case, the per-step computation requires a few million floating-point operations, which is negligible for a modern processor relative to the 2.5 s stimulation interval ($\lambda_{online}$). To measure this, we benchmarked the complete per-step closed-loop decision (GPR interpolation with posterior variance, the neural-network forward pass, and the sampler) on an Intel^®^ Xeon^®^ Platinum 8375C (Intel Corporation, Santa Clara, CA, USA) (single-threaded, for a conservative estimate), averaged over 500 iterations and swept over the number of observed colors $n_{o}$. Table A1 summarizes the results:

**Table A1:** Latency due to GPR, NN, and sampler modules.

$n_{o}$ (Colors Observed)	GPR Latency (ms)	NN Latency (ms)	Sampler Latency (ms)	Total Latency (ms)	Total Latency (% of $\lambda_{\mathrm{online}}$)
1	0.23	0.22	0.76	1.21	0.05
98 (online maximum)	8.36	0.26	0.91	9.53	0.38
168 (full grid)	21.3	0.35	1.19	22.84	0.91

At the online maximum of $n_{o}$ = 98 observed colors, a complete update takes 9.5 ± 0.2 ms (about 0.38% of the 2.5 s stimulation interval). Even when all 168 colors are observed, the latency is only 22 ms (<1%). Because this computation executes during the 0.5 s inter-stimulus interval, it introduces no perceptible delay for the participant. We note that the neural network used for this timing benchmark was initialized with random weights rather than the trained model; since forward-pass latency depends only on the network architecture and not on the weight values, this does not affect the reported timings.

## Appendix C. Assumption of Normality

The multivariate-normal model and its log-transform were validated in our prior work [28]. We confirm those findings here through analysis of Q-Q plots for the 10 participants in the offline study, assuming a λ = 2.5 s online integration window, for both hypotheses (Figure A2). For each of the eight subbands, we pooled the within-participant-centered log-features (N = 9460 per subband) and assessed normality.

The log-features are close to Gaussian, and $H_{1}$ is nearly perfectly normal. The multivariate $\chi^{2}$(8) Q-Q plot shows the bulk of the distribution (the lower ∼90–95% of observations, where almost all data lie) falling tightly on the reference line, with a mild heavy-tail departure confined to a small number of extreme trials, slightly more pronounced under $H_{0}$.

## Appendix D. Ablation Testing

We conducted a module-level ablation study to isolate the contribution of each component to MetaIDNet’s overall performance. The study compares the following variants, progressively enabling one module at a time, evaluated with the same leave-one-out cross-validation and sum of squared errors (SSE) metric used in the main results of the paper:

**Table A2:** Summary of evaluated MetaIDNet variants.

Variant	Feature Extraction	Feature Translation	Prediction and Denoising	Sampling
Norton et al. (baseline)	CCA	None	None	Exhaustive
fbCCA	fbCCA	None	None	Exhaustive
CDM (no GPR)	fbCCA	CDM	None	Exhaustive
fbCCA + GPR	fbCCA	None	GPR	Exhaustive
CDM + GPR	fbCCA	CDM	GPR	Exhaustive
GPR + Random	fbCCA	CDM	GPR	Random
GPR + Max-Variance	fbCCA	CDM	GPR	Max variance
MetaIDNet (full)	fbCCA	CDM	GPR	Neural network

Feature Extraction—Replacing CCA with fbCCA reduces SSE by 13.7% (from 122.7 to 105.9 at 12.6 min corresponding to λ = 4 s), consistent with our prior work.

Feature Translation (CDM) and Prediction and Denoising (GPR)—These two modules are the largest contributors to the improvement in performance of MetaIDNet compared with the comparison modules. Applying GPR is beneficial regardless of the underlying feature; it reduces SSE by 21.7% on fbCCA features (from 105.9 to 83.0 at 12.6 min, corresponding to λ = 4 s) and by 24.8% on CDM features (from 99.2 to 74.5 at 12.6 min, corresponding to λ = 4 s). Adding CDM on top of fbCCA + GPR lowers SSE by an additional 10.2% (from 83.0 to 74.5 at 12.6 min, corresponding to λ = 4 s).

Neural Network Sampler—To isolate the contribution of the learned sampling policy, we hold the feature extraction, translation, and denoising modules fixed (all variants use fbCCA + CDM + GPR) and vary only the sampling strategy. At a fixed 5-min assessment, the neural network reduces SSE by 13.8% relative to random sampling (from 108.3 to 93.3) and by 12.1% relative to a max-variance (uncertainty-based) active-learning policy (from 106.2 to 93.3).

Summary—The ablation confirms that each module contributes a distinct and necessary share of the overall performance: feature extraction and translation improve the per-color signal, GPR denoising and interpolation provide the largest accuracy gain, and the neural network sampler is what enables MetaIDNet to retain accuracy while sampling far fewer colors.

## Appendix G. Online SSE Under an Independent Ground Truth

Online SSE was compared against a fully independent reference —the offline exhaustive ground truth was collected in separate sessions and does not depend on MetaIDNet’s online sampling. This was possible for the four participants who completed both experiments (S1, S6, S7, S9); the remaining online participants (S11-S14) did not complete the offline protocol.

**Table A3:** Comparison of SSE for the three BCI-based CV assessment systems. Results are the average of the data from the four participants who completed both the offline and online experiments (i.e., S1, S6, S7, and S9).

	Norton et al.	fbCCA-Based	MetaIDNet
Original y*	175	149	91
Independent y* (offline exhaustive)	273	255	227
